# MicroRNAs in inflammatory lung disease - master regulators or target practice?

**DOI:** 10.1186/1465-9921-11-148

**Published:** 2010-10-28

**Authors:** Irene K Oglesby, Noel G McElvaney, Catherine M Greene

**Affiliations:** 1 Respiratory Research Division, Department of Medicine, Royal College of Surgeons in Ireland, Beaumont Hospital, Dublin, Ireland

## Abstract

MicroRNAs (miRNAs) have emerged as a class of regulatory RNAs with immense significance in numerous biological processes. When aberrantly expressed miRNAs have been shown to play a role in the pathogenesis of several disease states. Extensive research has explored miRNA involvement in the development and fate of immune cells and in both the innate and adaptive immune responses whereby strong evidence links miRNA expression to signalling pathways and receptors with critical roles in the inflammatory response such as NF-κB and the toll-like receptors, respectively. Recent studies have revealed that unique miRNA expression profiles exist in inflammatory lung diseases such as cystic fibrosis, chronic obstructive pulmonary disease, asthma, idiopathic pulmonary fibrosis and lung cancer. Evaluation of the global expression of miRNAs provides a unique opportunity to identify important target gene sets regulating susceptibility and response to infection and treatment, and control of inflammation in chronic airway disorders. Over 800 human miRNAs have been discovered to date, however the biological function of the majority remains to be uncovered. Understanding the role that miRNAs play in the modulation of gene expression leading to sustained chronic pulmonary inflammation is important for the development of new therapies which focus on prevention of disease progression rather than symptom relief. Here we discuss the current understanding of miRNA involvement in innate immunity, specifically in LPS/TLR4 signalling and in the progression of the chronic inflammatory lung diseases cystic fibrosis, COPD and asthma. miRNA in lung cancer and IPF are also reviewed.

## Introduction

Inflammatory lung diseases encompass a range of conditions which can be divided into acute and chronic diseases. Acute inflammatory lung diseases, for example pneumonia, in large part are triggered by environmental stimuli without the contribution of a genetic factor. Airway disorders associated with persistent inflammation such as cystic fibrosis (CF), chronic obstructive pulmonary disease (COPD) and asthma are classed as chronic conditions and are influenced by a combination of environmental, genetic and epigenetic components [1]. Infections causing severe systemic inflammation, depending on the magnitude of the initial stimulus can be short lived dissipating within days or weeks [2], whereas the sustained inflammation observed in CF, COPD and asthma can elicit deleterious effects leading to lung tissue damage, a constant need for medication and poor quality of life. Dysregulated inflammation is also observed in lung cancer and IPF and collectively with CF, COPD and asthma these conditions account for a huge percentage of deaths worldwide and hence there is an increasing need for the development of new therapies.

Elements of signalling pathways activated during the progression of chronic inflammation in the lung represent a vast area for miRNA studies. Although still in its infancy the involvement of miRNA in inflammatory lung diseases is becoming rapidly apparent (Table 1). Several investigators have implicated negative feedback control of inflammation via both regulators of inflammatory signalling and miRNA induction which we refer to later on in the review. We also discuss the biogenesis of miRNA and their role in the acquired and innate immune response highlighting specifically those linked to LPS/TLR4 signalling. We describe inflammatory lung disease in CF, COPD and asthma and explore what is currently known about miRNA and its role in the pathogenesis of these debilitating conditions along with a brief look at miRNA in lung cancer and IPF.

**Table 1:** Selected miRNA implicated in inflammatory lung disease

miRNA	Tissue/Cell Type	Species	Condition/treatment	Validated Target Genes	References
Let-7d	Lung biopsies	Human	IPF	HMGA2	[124]

miR-21	Whole lung	Mouse	Asthma	IL-12p35	[94]
	Macrophages				
	Dendritic cells				
	Lung biopsies	Human	IPF	Smad, Smad7	[125]

miR-126	Primary bronchial epithelial cells	Human	CF	TOM1, Tollip	[112]
	Lower airway tissue	Mouse	Asthma	OBF.1	[120]

miR-133a	Bronchial smooth muscle cells	Human	Asthma	RhoA	[97]

miR-146a	Lung alveolar epithelial cells	Human	IL-1β	IL-8, RANTES	[67]

miR-218	Primary bronchial epithelial cells	Human	CSE	MAFG	[87]

miR148a,b, Human miR-152	Primary bronchial epithelial cells		Asthma	HLA-G	[24]

# Let-7c miR-34c miR-222	Whole lung	Rat	CSE	ND	[86]

miR-26b, 27a, miR-31*, 96,	Primary bronchial epithelial cells	Human	DEP	ND	[88]

miR-135b,274a,					
miR-338-5p, 494,					
miR-513a-5p, b, c,					
miR-923					

Let-7a, b, f,	Whole lung	Mouse	CSE	ND	[85]
miR-26a, 30b, c,					
miR-34b, 99b, 122a,					
miR-124a, 125a, b, 140,					
miR-192, 431					

CSE; cigarette smoke extract, ND; not determined, DEP; diesel exhaust particulate, # 3 of 24 down-regulated miR's validated by qRT-PCR

### miRNA background

The discovery of miRNA is considered one of the major breakthroughs of the last decade. However they were probably first mentioned in the 1960's when Britten and Davidson proposed the existence of "activator" RNAs transcribed from redundant genomic regions [3]. In 1993 the first miRNA *lin-4 *was discovered in *C.elegans *[4] and it wasn't until eight years later that again in *C.elegans *the highly conserved *let-7 *was found to be crucial for developmental timing [5]. The term microRNA was coined when a large number of these small RNAs with potential regulatory roles were discovered in 2001 [6]. The following year established the presence of miRNAs in plants [7]. Subsequently miR-15 and miR-16 were shown to be deleted or down-regulated in chronic lymphocytic leukemias [8] suggesting miRNA involvement in cancer in humans.

MiRNAs are small, endogenous RNAs, approximately 20-25 nucleotides long which negatively regulate gene expression at a post-transcriptional level. They exert direct effects on gene expression by inducing mRNA degradation or translation inhibition and recently have been reported to act by indirect means through global effects on methylation or targeting of transcription factors [9,10]. Up-regulation of gene translation by miRNAs has also been reported albeit to a lesser extent, where miR-369-3 for example, has been shown to switch from mRNA repression to activation in quiescent cell populations [11]. Interest in miRNAs over the past decade has increased exponentially uncovering their importance in biological processes such as development, proliferation and apoptosis. Relatively few miRNAs have been studied in detail and hence the precise biological relevance of the majority remains to be uncovered. It is estimated that up to one third of the human genome may be subject to regulation by miRNAs. Expression levels vary greatly among tissues and it is believed that dysregulation of miRNAs can contribute to disease pathology [12].

#### miRNA - origin, processing and target selection

**Figure 1:**
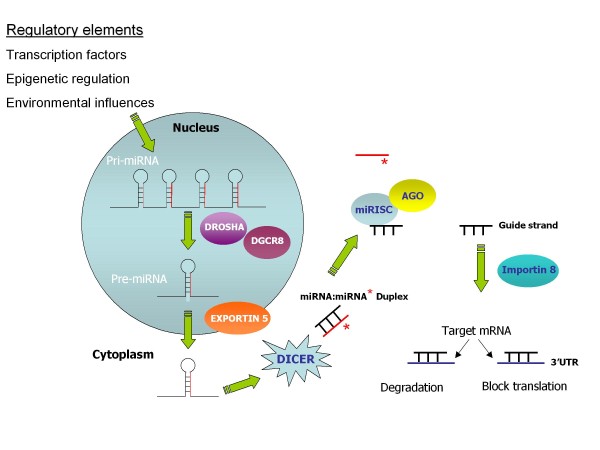
**Representation of miRNA induction and biogenesis**. Induction of miRNA expression occurs via transcription factors and other regulatory influences. Primary miRNA transcripts (pri-miRNA) produced in the nucleus are generally processed by Drosha and DGCR8 into pre-miRNA hairpin structures and transported to the cytoplasm by Exportin 5. The pre-miRNA is processed by Dicer into a miRNA duplex where the mature miRNA guide strand is incorporated into a miRNA-induced silencing complex (miRISC) and the miRNA* strand is degraded. Incorporation into miRISC is facilitated by AGO proteins. Importin 8 aids subsequent transport of the miRNA to the target mRNA where binding to the 3'UTR occurs resulting in translational repression or degradation.

Primary miRNA (pri-miRNA) originate in the nucleus as single long transcripts up to 1000 nucleotides long which can be processed by the RNase III enzyme Drosha and the RNA binding protein DGCR8 into pre-cursor hairpin structures ~ 70-100nt long termed "pre-miRNA" [13-15]. Transport to the cytoplasm is via Exportin 5 where the pre-miRNA is further processed by Dicer into a miRNA duplex consisting of the mature miRNA and the so called miRNA* strand (which is generally degraded) [16]. Incorporation of the mature miRNA into a miRNA-induced silencing complex (miRISC) is facilitated by Argonate protein involvement and transport to the target mRNA has recently been reported to involve importin 8 [17] (Figure 1). Single miRNA can exist in introns and exons of so called host genes, whilst certain groups of miRNAs are present in clusters in the genome for example the miR-17-92 family. Within each miRNA there exists a 2-8 nucleotide "seed region" thought to be critical for target selection [18]. Mature miRNAs use this seed region to bind selectively to miRNA recognition elements (MRE) within the 3' untranslated region (3'UTR) of target mRNAs. Different target genes may contain several MREs and therefore be regulated by numerous miRNAs. The number of and distance between MREs are considered important for the biological activity of miRNAs. Targeting and subsequent repression of transcripts may also occur at the 5'UTR with equal efficacy [19]. However, the majority of studies have revolved around determination of miRNA:mRNA interactions at the 3'UTR.

### miRNA regulation

The matter of miRNA regulation and expression has been subject to much speculation with multiple levels of control being identified in recent studies. O'Connell *et al *describe three levels of control, namely at the stages of "(i) transcription, (ii) processing and (iii) subcellular localisation" [20]. Stage (i) includes induction of miRNA expression by transcription factors in response to inflammatory stimuli and cellular stresses, stage (ii) impaired processing may be due to dicer inhibition [21] or post-transcriptional modifications [22] and finally stage (iii) is where miRNA can localise to stress granules and p-bodies; a process which is poorly understood at this time. Epigenetic mechanisms controlling miRNA expression have been described whereby down-regulation of miR-126 for example can be induced by inhibitors of DNA methylation and histone deacetylation [23]. Alterations in miRNA function can also occur as a result of single nucleotide polymorphisms (SNPs). Tan *et al *reported compromised binding of miR-148a, -148b and -152 to the asthma susceptibility gene HLA-G due to the presence of a polymorphism in the 3'UTR [24].

The majority of miRNA-induced changes in gene expression are subtle exerting a modest 2-3 fold repression of targets which can hamper elucidation of miRNA function [25]. However, the most important miRNA-regulated effects appear to occur as a result of miRNA down-regulation or deletion. For example, miR-126 was shown to be essential for maintenance of vascular integrity and angiogenesis *in vivo *whereby targeted deletion (mice) or knockdown (zebrafish) resulted in partial embryonic lethality or leaky vessels and haemorrhaging, respectively [26,27]. MiR-126 and miR-335 are specifically lost as breast cancer cells move towards metastasis and consequently these miRNAs have been suggested to function as metastasis suppressor miRNAs in human breast cancer. Their re-introduction into breast cancer cells *in vivo *led to reduced tumor growth, proliferation and inhibition of invasion [28]. It is also evident that changes in gene expression in one cell type may not occur in another, as illustrated in zebrafish by Mishima *et al *[29], where miR-430 repression of a subset of target genes in somatic cells had no effect on the same genes in primordial germ cells. This supports evidence for cell specific miRNA participation in different pathways and also draws attention to the regulation of miRNA by multiple mechanisms. One such miRNA regulatory mechanism recently described opens a potentially vast area of study whereby previously ignored "Junk" pseudogenes may act as decoys for miRNA thereby preserving expression of their functionally relevant counterpart. Poliseno *et al *reported that the pesudogene PTENP1 of tumor suppressor PTEN, presents a conserved region in its 3'UTR to which miRNA bind, leading to de-repression of PTEN and enhanced tumor suppressor activity [30]. Recent work in *C.elegans *and plants have uncovered proteins that regulate miRNA turnover [31,32], however this has not yet been demonstrated in mammals.

### miRNA - immune response and TLR signalling

#### Acquired immunity

This arm of the immune response mediated by T and B lymphocytes is probably one of the best characterised developmental systems in mammals. Many investigators have described an integral role for miRNA in acquired immunity and these studies have been extensively reviewed [20,33-36]. The importance of miRNA in both T and B cell development has been demonstrated via disruption of miRNA processing machinery. For example, T-cell specific deletion of dicer results in reduced T cell numbers and a progression towards autoimmune pathology [37] and in B-cells dicer knockout impairs pro- to pre-B cell progression almost completely accompanied by increased level of the pro apoptotic protein Bim [38]. MiR-150 has been reported to block production of mature B cells [39] via dysregulation of transcription factor C-myb which elicits critical control during multiple steps of lymphocyte differentiation [40]. Over-expression of miR-181 causes a marked in increase in B cells numbers with no effect on T cells or myeloid cells [41] and can regulate sensitivity of T cell receptor signalling to antigens [42]. The importance of miR-155 in maintenance of a normal immune response was highlighted by Rodriguez *et al *who observed that mice deficient in miR-155 display diminished B and T cell responses and favored differentiation of helper T (T_H_) cells towards a T_H_2 response [43].

#### Innate immunity

The innate immune response is a complex, highly organised first line of host defense initiated upon exposure to invading pathogens and allergens. Carefully balanced resolution of inflammation triggered by infection is critical to the prevention of chronic disease states. In the airways cellular components such as alveolar macrophages, neutrophils and epithelial cells have key functions in this process [44]. Once thought to be a non-specific response to keep infection at bay while the adaptive immune system prepared appropriately it is now known to be a prerequisite for initiation of several adaptive immune response components. Toll like receptors (TLRs) are a family of pattern recognition receptors that play an essential role in innate immunity by responding to microbial antigens such as lipopolysaccharide (LPS) and initiating signalling cascades that culminate in pro-inflammatory gene expression, principally via activation of the transcription factors NFkB and the IRFs [45-47]. However, whilst crucial for pathogen clearance, under or over-exuberant TLR signalling can be deleterious to the host leading to the pathogenesis of chronic inflammatory conditions [48,49]. In the lung TLRs are expressed by immune cells and also epithelial cells [50]. A number of reports have provided evidence of miRNA involvement in immune cell development and in regulation of the innate immune response in part via establishment of a link between TLR signalling, miRNA expression and activation of inflammatory pathways [33-36,51-53]. Both bacterial and viral ligands of the TLRs can induce the expression of several miRNA as can inflammatory cytokines produced as a direct result of TLR stimulation.

#### LPS/TLR4 signalling

**Figure 2:**
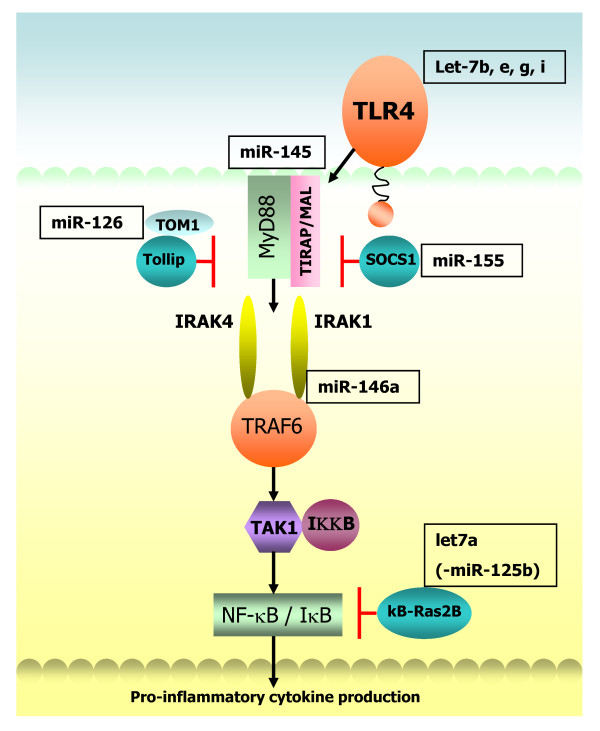
**Selection of miRNA implicated in the TLR4 signalling pathway**. TLR4 signals via MyD88 and TIRAP/MAL to IRAKs 1 and 4. The TOM1/Tollip complex and SOCS1 are negative regulators of this pathway. The signal is transduced via TRAF6, TAK1 and IKK leading to activation of NF-κB via dissociation of IκB. κB-Ras2B is an IκB inhibitor. miRNA targeting components of TLR4 signalling are shown in boxes. MiR-145 [126].

Of the TLRs characterised to date, TLR4 in particular has been the subject of the majority of studies where a firm link between miRNA expression and TLR4 signalling pathways is now recognised, (depicted in Figure 2). The vast majority of studies have been performed in macrophages (murine and human, *in vitro *and *in vivo*) and to a lesser extent, neutrophils, PBMCs and epithelial cells. LPS, one of the major ligands for TLR4 has been associated with induction of several miRNA including miR-155, -146, -9 and -21 to name a few, which were shown to target SHIP1, IRAK1/TRAF6, NF-κB1, and tumor suppressor PDCD4 respectively [54-57]. Expression of miR-155 can also be induced by additional TLR ligands and a plethora of pro-inflammatory cytokines [53] and in the context of inflammatory lung disease mice deficient in miR-155 display immunodeficiency and substantial airway remodelling [43]. Notably, the position of the Bic/miR-155 gene in humans is located on chromosome 21q21 mapping to a region of asthma and pollen sensitivity [58,59]. Up-regulation of miR-9 expression shown to occur upon treatment with TLR2, 4, 7 and 8 agonists, IL-1β and TNF-α but not IFNγ was the only miRNA found to be differentially expressed in both human neutrophils and monocytes following stimulation with LPS [54].

Direct regulation of TLR4 by let-7i in human bilary epithelial cells was reported by Chen *et al *who showed that in the presence of LPS, let-7i expression is reduced in a MyD88/NF-κB dependent manner accompanied by a concomitant increase in TLR4 protein. Two other members of the let-7 family detected in these cells (let-7b and let-7g) are also predicted to target TLR4 [60]. Contrary to this, let-7a has been shown to be induced following LPS stimulation in primary human macrophages [61]. The same study demonstrated an LPS-induced decrease in miR-125b whereby the net effect of LPS regulation of these miRNA resulted in inhibition of κB-Ras2, an inhibitor of IκB. Pre-treatment with 17β Estradiol abrogated the effects of LPS and was shown to suppress NF-κB activation through induction of κB-Ras2 via regulation of let-7a and miR125b, suggesting estrogen mediated regulation of miRNAs may be an important factor in control of inflammation [61]. Let-7a induction was observed to be NF-κB dependent whereas the LPS-induced decrease in miR-125b expression was independent of this pathway. Supporting these findings Androlidaki *et al *reported that LPS suppression of miR-125b is dependent on Akt signalling in murine macrophages both *in vitro *and *in vivo *[62].

#### Negative feedback control of inflammation

Various means of intracellular and extracellular negative control of inflammation for the maintenance of a balanced response to TLR signalling exist. Endogenously, these include decoy molecules that compete for binding to TLRs such as soluble TLRs (sTLRs) [63] and a broad spectrum of intracellular molecules ranging from nuclear receptors to proteases, kinases, transmembrane proteins, ubiquitin-modifying and adaptor proteins [64]. Emerging data on the role of miRNA in the immune response indicates that their induction and regulation may play an important role in the termination of inappropriate signalling. For example TNF-α mediated induction of miR-31 and miR-17-3p target endothelial adhesion molecules E-selectin and ICAM-1 respectively, which are both induced by TNF-α thereby providing negative feedback control of inflammation [65]. Another recent study to examine the effects of IL-10 (a potent anti-inflammatory cytokine poorly expressed in asthma) on TLR4 signalling demonstrates that IL-10 can suppress LPS-induced miR-155 expression in a STAT3-dependent manner leading to an increase in SHIP1 in bone marrow derived cells and in human PBMCs. LPS is known to induce IL-10 and negatively impact on this pathway by switching off the inflammatory response, so again this suggests a feedback loop whereby induction of IL-10, promoted by LPS, keeps LPS-induced miR155 expression in check. IL-10 was found to have no effect on two other LPS induced miRNA - miR-146a and miR-21 [66].

A role for miR-146 in "fine tuning" of the innate immune response was described by Taganov *et al *when they found that LPS-induced increased expression of miR-146a was driven by NF-κB in the monocytic cell line THP-1 and negatively regulates IRAK1 and TRAF6, two key adaptor molecules downstream of TLR and IL-1R signalling, again illustrating a negative feedback control loop [57]. Increased expression of miR-146a was also induced upon treatment with TNF-α and IL-1β to a lesser extent and stimulation with other TLR ligands revealed up-regulation in response to TLR2 and TLR5 activation but not by TLR3, 7 and 9 ligands [57]. In human alveolar epithelial cells stimulation with IL-1β elicited a rapid increase in miR-146a expression and to a lesser degree miR-146b, corresponding to a decrease in the expression of pro-inflammatory chemokines IL-8 and RANTES, which the authors again propose as negative feedback control of inflammation. Incidentally neither IL-8 nor RANTES are predicted to contain binding sites for miR-146a. Examination of miR-146 expression in primary bronchial epithelial cells revealed a comparable increase in IL-1β-induced levels of miR-146a however miR-146b was not detected in these cells [67].

### miRNA and inflammatory lung disease

#### miRNA in lung cancer - utility as prognostic tools

Lung cancer is the leading cause of cancer-related deaths worldwide and to date the role of miRNA in lung cancer has been studied and reviewed extensively in comparison to the other lung diseases. Lung cancer can be broadly classified into small cell (SCLC) and non-small cell lung cancer (NSCLC), the latter being more common can be sub-classified into squamous and adenocarcinoma. Approximately 15-20% of human cancers are heavily associated with inflammation [68] where the involvement of NF-κB activation has been well documented [69]. Briefly, many miRNA identified as either tumor suppressors or oncogenes in lung cancer are also reported to be involved in the immune response, for example the let-7 family and the miR-17-92 cluster, respectively. Reduced expression of the let-7 family members in human lung cancer has been correlated with poor survival [70] and delivery of exogenous let-7 to a mouse model of NSCLC resulted in reduction of tumor load supporting a role for this miRNA as a tumor suppressor [71]. The miR-17-92 cluster over-expressed in B cell lymphomas [72,73] was found to be markedly increased in lung cancer by Hayashita *et al *in particular in those with SCLC histology [74] and thus has been classified as an onco-miR. Down-regulation of miR-126, also a proposed tumor suppressor correlated with reciprocal expression of vascular endothelial growth factor (VEGF) in eight lung cancer cell lines and was shown to reduce tumor growth in a xenograft mouse model [75].

With the detection of miRNA in blood cells and serum as well as in tissue their value as potential diagnostic and prognostic tools in cancer is emerging. Already in a study of individuals with NSCLC, a five miRNA signature (consisting of 2 protective miRNA - miR-221 and let-7a and 3 associated with poor prognosis miR-137, -372 and -182*) has been identified for prediction of treatment outcome [76]. MiRNA have demonstrated remarkable stability in sputum where a combination of miR-21, -486, -375 and -200b were shown to distinguish lung adenocarcinoma from normal subjects with >80 and 90% sensitivity and specificity respectively [77] and in serum where high levels of miR-141 discriminated individuals with prostate cancer compared to controls [78]. Classification of lung squamous cell carcinoma versus adenocarcinoma was also reported based purely on detection of high levels of miR-205 found in the former which are not present in the latter [79].

#### Chronic obstructive pulmonary disease (COPD)

COPD is a disease predominantly induced by cigarette smoking and/or chronic exposure to irritants. Emphysematous lung disease in alpha-1 antitrypsin (α1-AT) deficient individuals can also come under this heading. Hallmarks of COPD include a progressive obstruction of the airflow, chronic inflammation in the lungs, shortness of breath, chronic cough and phlegm production [80,81]. COPD is classified as the 4^th ^leading cause of death in the US currently and is predicted to increase to 3^rd ^place by 2020 [82]. Both chronic inflammation in the airway and systemic inflammation (potentially resulting from an excess of pro-inflammatory mediators entering systemic circulation) have been attributed to the pathogenesis of COPD [83]. In particular, during exacerbations elevated levels of numerous inflammatory markers such as interleukin 6 (IL-6), IL-8, TNF-α and circulating activated neutrophils have been reported [83]. These high levels of primed neutrophils correlate with increased oxidative stress and the severity of exacerbation [84].

The expression of miRNA in mouse and rat lungs exposed to cigarette smoke extract (CSE) has been studied by Izotti *et al *[85,86] where down-regulation of miRNA was observed predominantly in both models. Out of 484 miRNA analysed, 126 were down-regulated by at least 2-fold and 24 ≥ 3-fold in smoke exposed rats, with 15 down-regulated in mice compared to just one and none up-regulated in the same animals. This is consistent with the results of miRNA expression profiling in human bronchial epithelium of 10 smokers versus 9 never smokers [87]. Twenty three miRNA were significantly down-regulated and only 5 up-regulated out of a total of 467 analysed in the smokers [87]. *In silico *analysis of miRNA altered in mice exposed to CSE linked their biological roles to a variety of functions including development of airway epithelial cells, stress responses, formation of pulmonary surfactant and inflammation [86]. Many miRNA involved in activation of the NF-κB pathway such as miR-30, miR-146, miR-132 and miR-155 were down-regulated by CSE in rodents [86]. Contrary to the CSE studies primary human bronchial epithelial cells exposed to diesel exhaust particles (DEP) exhibited a predominant up-regulation (n = 130) compared to down-regulation (n = 67) of miRNAs. Like cigarette smoke, DEP has been linked to pulmonary inflammation, increased risk of cancer and exacerbations in asthma and COPD [88]. Bioinformatic analysis to determine the biological relevance of miRNA altered by DEP exposure generated molecular networks enriched for inflammatory pathways which correlated to IL-8, NF-κB and CXCR4 signalling pathways. MiR-494 (increased ≥ 1.5 fold upon DEP exposure) was predicted to have important roles in NF-κB and virus activated signalling [88].

#### Asthma

Asthma is a widespread complex disease affecting approximately 300 million people worldwide [89]. Its pathogenesis can be attributed to both genetic factors and exposure to environmental triggers at various stages of development ranging from pre-natal maternal exposure to allergens through to occupational exposure in adulthood for example [90]. Asthma is characterised by chronic inflammation caused by persistent infiltration of eosinophils and mast cells, an abnormal T helper-2 (T_H_2) response and the downstream actions of their secreted cytokines. The T_H_2 cytokines IL-4, 5, 9 and 13 have all been linked to airway hyper-responsiveness (AHR), mucus hypersecretion, eosinophil infiltration and elevated levels of IgE [91,92]. Structural changes leading to airway remodelling combined with chronic inflammation give rise to its hallmarks - reversible AHR and airway obstruction [93].

Focus on the role of miRNA in asthma has increased recently with a number of studies in the past year reporting differential expression of miRNA in various models of asthma. In a study of 579 miRNAs in bitransgenic mice expressing IL-13 (a key T_H_2 derived cytokine) in a lung specific manner, 21 were differentially expressed upon induction of experimental asthma following treatment with doxycycline [94]. The most up-regulated miRNA was miR-21, with miR-1 being the most down-regulated. These observations were replicated in two other separate mouse models of experimental asthma [94]. All three asthma models shared similar phenotypes including AHR, mucus production and T_H_2 eosinophilic inflammation and highest expression levels of miR-21 were observed in macrophages and dendritic cells. Independently, Lu *et al *showed IL-4 transgenic mice demonstrated increased expression of miR-21. IL-13-induced expression of miR-21 was shown to be dependent on the receptor IL-13Rα1, with allergen induced expression mostly independent of this receptor. Up-regulation of miR-21 correlated with down-regulation of its predicted target IL-12p35 which was confirmed as a molecular target using a luciferase reporter system. Interestingly IL-12 derived from macrophages and dendritic cells is involved in T_H_1 polarisation in the adaptive immune response [95,96], suggesting that miR-21 may potentially regulate a number of processes involved in allergic airway inflammation [94]. Another study featuring IL-13 influences on miRNA expression was performed by Chiba *et al *[97]. The monomeric GTP-binding protein RhoA has been associated with contraction of smooth muscle, whereby its over-expression in an animal model of allergic bronchial asthma augmented contraction of bronchial smooth muscle [98,99]. RhoA over-expression correlated with down-regulation of miR-133a in human bronchial smooth muscle cells (hBSM) treated with IL-13 and in bronchial tissue from sensitised BALB/c mice repeatedly challenged with ovalbumin [97]. This data suggests that IL-13, one of the major up-regulated cytokines in asthmatic airways, may play a role in AHR induction in part through repression of miR-133a and inducing increased expression of RhoA [97].

Surprisingly, expression profiling of 227 miRNA in airway biopsies from mild asthmatics before and after treatment with the inhaled corticosteroid budesonide and also compared to healthy non-asthmatics revealed no differences in miRNA expression [100]. However, analysis of a range of cell types found within human lung (isolated from a 5 donor pool) including macrophages, airway smooth muscle cells, epithelial cells and fibroblasts demonstrated miRNA profiles specific to particular cell types. The majority of highly expressed miRNA were contained within alveolar macrophages compared to other cell types, in particular miRs -30a-5p, -92, -142-3p, -146b, -191, -223 , -320 and -342. In order to explain this, the authors proposed that differential miRNA expression may occur in mild asthmatics possibly within specific cells types which could have been masked in the airway biopsies tested due to the presence of multiple cell types. Potentially in more severe cases of asthma, differential miRNA expression might be more evident [100].

#### Cystic fibrosis (CF)

CF is one of the commonest lethal genetic diseases in Caucasians with approximate incidences of 1:3500 in Europe and North America. CF is also evident in African and Asian populations albeit to a much lesser degree (1:5000 - 1:20,000) [101]. Despite significant advances in treatment regimes CF remains a condition for which there is no effective cure with >90% of deaths occurring as a result of respiratory failure [102]. Following establishment of CF as a disease with altered chloride ion transport the gene responsible for the cause of CF (CF transmembrane conductance regulator - CFTR) was discovered in 1989 [103,104]. There have been over 1400 disease-causing mutations identified to date in the CFTR gene and these can be generally grouped into six classes based on cellular phenotype [105]. Δ508 which falls into class II is the most prevalent mutation worldwide [106].

CF lung disease is characterised by chronic infection, exaggerated intra-pulmonary protease levels e.g. neutrophil elastase (NE) and neutrophil dominated airway inflammation. Impaired chloride ion channel secretion results in a build up of thick sticky mucus and reduced mucocilary clearance leading to bacterial colonisation with bacteria such as *Pseudomonas aeruginosa*. The gram-negative bacterial cell wall component LPS and IL-1β, which bind to Toll-Like Receptor 4 (TLR4) and the IL-1 Type-I receptor (IL-1RI), respectively, play a pivotal role in this process. These agonists can activate the innate immune response culminating in pro-inflammatory gene expression leading to infiltration of large numbers of neutrophils contributing to sustained endobronchial inflammation and tissue damage in the CF lung. In the context of CF airway epithelial cells both the IL-1RI and TLRs have been shown to promote pro-inflammatory gene transcription following stimulation with their cognate agonists [107,108]. For example, in airway epithelial cells of non-CF and CF origin triacylated lipopeptide, LPS or unmethylated CpG DNA can induce IL-6, IL-8 and TNF-α production via TLRs 2, 4 and 9 [107]. Similarly, IL-1β can up-regulate production of a plethora of pro-inflammatory cytokines [108]. Thus IL-1RI and/or TLRs and their signalling intermediates represent potential therapeutic targets for CF. Accumulation of neutrophils with impaired function, their subsequent degradation and release of intracellular contents including NE contributes greatly to sustained protease-antiprotease imbalance [109] and damage to structural, cellular and soluble components of the CF lung [110]. NE has also been shown to induce IL-8 expression via MyD88/IRAK/TRAF6 pathways in human bronchial epithelium [111].

We have previously reported differential expression of miRNA in CF whereby expression profiling of 667 different human miRNAs was performed on bronchial brushings taken from CF and non-CF individuals (n = 5 each) by qRT-PCR, [112]. Expression of 391 miRNAs was detected across all samples with 56 being down-regulated (Relative Quantification (RQ) ≤ 0.7) and 36 up-regulated (RQ ≥ 1.5) in CF versus non CF controls. Expression of miR-218 remained unchanged in the bronchial epithelium of CF versus non-CF individuals [112], however it was found to be the most significantly down-regulated miRNA in bronchial epithelial cells of smokers vs. non- smokers in a study by Shembri *et al *[87] providing support of the phenomenon of disease-specific expression of miRNA. Consistent with a report of miR-494 up-regulation in bronchial epithelial cells exposed to DEP [88] we also found this miRNA to be up-regulated in CF versus non-CF bronchial brushings [112]. MiR-126, discussed in the following section, was found to be significantly decreased in four of the five CF samples compared to controls. We are currently pursuing the role of a selection of miRNA and their predicted biological targets resulting from follow-up *in silico *analysis, with a view to uncovering previously unidentified regulatory mechanisms that may control changes in gene expression and direct the development of future therapeutic strategies, possibly directed at IL-1RI/TLR signalling, for this debilitating and fatal disorder.

#### miR-126 in CF and asthma - a key innate immune regulator?

MiR-126 is 21 nucleotides in length, located on chromosome 9q34.3 and is contained within intron 5 of its host gene epidermal growth factor like-7 (EGFL-7) [113]. It has previously been shown to have functional roles in angiogenesis [26,27], to be down-regulated in a number of malignancies [23,114] and to act as a tumor suppressor in breast cancer [28]. We selected miR-126 for further investigation following miRNA expression profiling in CF vs. non-CF bronchial brushings given that its expression is known to be highest in vascularised tissues such as the lung, heart and kidney [113,115,116] and as it has been shown to be present in bronchial epithelium [114]. We found down-regulation of miR-126 in CF bronchial epithelial cells correlated with a significant up-regulation of TOM1 mRNA when compared to their non-CF counterparts. TOM1 (target of Myb1) has been shown to interact with Toll interacting protein (Tollip), forming a complex to regulate endosomal trafficking of ubiquitinated proteins. TOM1 has also been proposed as a negative regulator of IL-1β and TNF-α induced signalling pathways [117-119]. Over-expression of miR-126 decreased TOM1 protein production in CF bronchial epithelial cells and decreased luciferase activity in a reporter gene containing the full length 3'UTR of TOM1 demonstrating direct targeting. Following stimulation with LPS or IL-1β, over-expression of TOM1 was found to down-regulate NF-κB luciferase activity. Conversely, TOM1 knockdown resulted in a significant increase in NF-κB regulated IL-8 secretion suggesting that miR-126 may have an important role in regulating innate immune responses in the CF lung.

Neither LPS nor IL-1β alone had any effect on miR-126 expression and similarly Harris *et al *showed miR-126 was not altered in response to TNF-α [113]. They reported miR-126 inhibits vascular cell adhesion molecule 1 (VCAM1) *in vitro *and proposed a regulatory role for miR-126 in inflammation, specifically in the vasculature. A role for miR-126 in the pathogenesis of allergic asthma was described by Mattes *et al *who showed increased expression levels of miR-16, -21 and -126 in the lower airway tissue isolated from a BALB/c mouse model of allergic asthma induced by house dust mite (HDM) challenge. Inhibition of miR-126 resulted in abrogation of the asthmatic phenotype as demonstrated by a reduction in AHR, inflammation, T_H_2 responses, mucus hypersecretion and eosinophil recruitment. They concluded that miR-126 expression is dependent on both the TLR4 and MyD88 pathways as they failed to detect its up-regulation in the airways of either TLR4 or MyD88 deficient mice. These mice failed to display any of the hallmark features of asthma despite repeated allergen challenge [120]. Further analysis of miR-126 inhibition in airway walls of HDM sensitised mice revealed significant up-regulation of Oct binding factor 1 (OBF.1), known to be a critical regulator of the transcription factor PU.1 which can negatively regulate T_H_2 responses and TLR4 expression through suppression of GATA3 [121,122]. Notably the suppression of T_H_2 responses occurred in the absence of a T_H_1 response, indicating specific targeting of the T_H_2 regulated inflammatory response by miR-126 in allergic lung disease [120]. Taken together with our data this supports an important role for miR-126 in the innate immune response of the lung.

#### Idiopathic pulmonary fibrosis (IPF)

IPF is a progressive disease of the lung interstituim characterised by epithelial cell injury, fibroblast proliferation and excessive accumulation of extracellular matrix proteins such as collagen within the lung parenchyma. This is usually accompanied by increased levels of the key fibrotic mediator transforming growth factor β (TGF-β) and other cytokines produced at the site of active fibrosis [123]. Evidence of miRNA involvement in the pathogenesis of IPF is just starting to emerge with a number of recent studies describing differential miRNA expression. Pottier *et al *compared miRNA expression profiles of normal lung fibroblasts with alveolar epithelial cells and found a selection of miRNA were preferentially expressed in fibroblasts. Expression of miR-155 was up-regulated in the presence of TNF-α and IL-β with a concomitant reduction of keratinocyte growth factor (KGF) in normal pulmonary fibroblasts leading to increased fibroblast migration via activation of caspase 3. Following treatment with TGF-β, miR-155 levels were reduced. In a mouse model of fibrosis miR-155 levels correlated with the degree of fibrosis [123]. Expression profiling of human IPF lung tissue biopsies compared with controls revealed differential expression of 46 miRNA. Of those, 18 were significantly down-regulated including miR-126, miR-203, several members of the miR-30 family and let-7d. Again TGF-β was found to decrease let-7d which correlated with an up-regulation of its predicted target HMGA2. Knock down of let-7d resulted in increased levels of collagen and alveolar septal thickening in mouse lungs *in vivo *and epithelial mesenchymal transition *in vitro *[124]. The oncogenic miR-21 was also observed to play a role in IPF whereby it was found to be up-regulated in human IPF samples and in the lungs of mice with bleomycin induced fibrosis. Enhanced miR-21 expression was observed in primary pulmonary fibroblasts treated with TGF-β. Inhibition of miR-21 decreased the severity of fibrosis whereas over-expression promoted TGF-β induced fibrotic activity [125].

## Conclusion

Intensive research over the past decade has rapidly advanced our knowledge of miRNA participation in numerous biological processes and disease states. It is clear that both appropriate miRNA regulation and inappropriate dysregulation forms a major part of mammalian development and disease progression respectively. In the early stages of miRNA discovery it was predicted that they could potentially regulate up to one third of the human genome which is probably largely underestimated with some target prediction databases suggesting thousands of potential targets for certain miRNA. One single miRNA has the capacity to regulate hundreds of targets and likewise individual target genes may be regulated by several miRNA depending on the amount of MREs they possess. Questions remain as to whether preferential binding of certain miRNA occurs, i.e. are some miRNA more dominant than others? A number of profiling studies have reported that the majority of differentially expressed miRNA in disease states are down-regulated [85-87]. We observed this to be the case in CF where almost twice as many miRNA were down-regulated compared to up-regulated versus non-CF controls. Upon *in silico *analysis for a number of target genes of particular interest to our laboratory we found several predicted to be regulated by an equal number of both up- and down regulated miRNA, suggesting possible maintenance of a balance of gene expression.

In summary, we have outlined some of the evidence identifying miRNA as key regulators of the innate immune response with particular reference to TLR4 signalling and negative feedback control of inflammation where both miRNA and transcription factors can regulate one another to maintain a stable cellular environment and whereby irregular expression can contribute to inflammatory disease progression. We discuss miRNA involvement in lung cancer and also describe the emerging role of miRNA in the progression of inflammatory lung diseases such as CF, COPD, asthma and IPF. The dysregulation of miRNA in chronic inflammatory lung disease represents an exciting possibility for therapeutic intervention however the development of strategies harnessing miRNA expression for treatment of these diseases is at an early stage and will take some time to optimise delivery, stability and specificity. Targeting miRNA expression in the lung may represent a realistic therapeutic intervention as aerosolisation enhances drug delivery to the site of action and decreases systemic exposure of the patient to the therapy, thereby reducing off-target effects. However the nucleic acid-based molecules used for miRNA modulation are large negatively charged moieties and these physicochemical properties limits intracellular delivery to their sites of action. In the meantime several challenges remain for investigators to fully elucidate the functional relevance of miRNA: mRNA interactions, to assess the impact of target gene regulation by several miRNA to establish if preferential binding/synergistic repression exists and also to establish the mechanism for clearance of miRNA from the cell, all of which will help define miRNA regulation in complex cellular systems and facilitate the development of specific therapeutic approaches.

## Competing interests

The authors declare that they have no competing interests.

## Authors' contributions

IKO and CMG drafted and revised the manuscript. IKO, NMcE and CMG read and approved the final article.
